# Supplementary figures and images for: Duplex Proximity Sequencing (Pro-Seq): A method to improve DNA sequencing accuracy without the cost of molecular barcoding redundancy
Source: PLoS One. 2018 Oct 2;13(10):e0204265. doi: 10.1371/journal.pone.0204265 (PMC6168144; doi:10.1371/journal.pone.0204265)

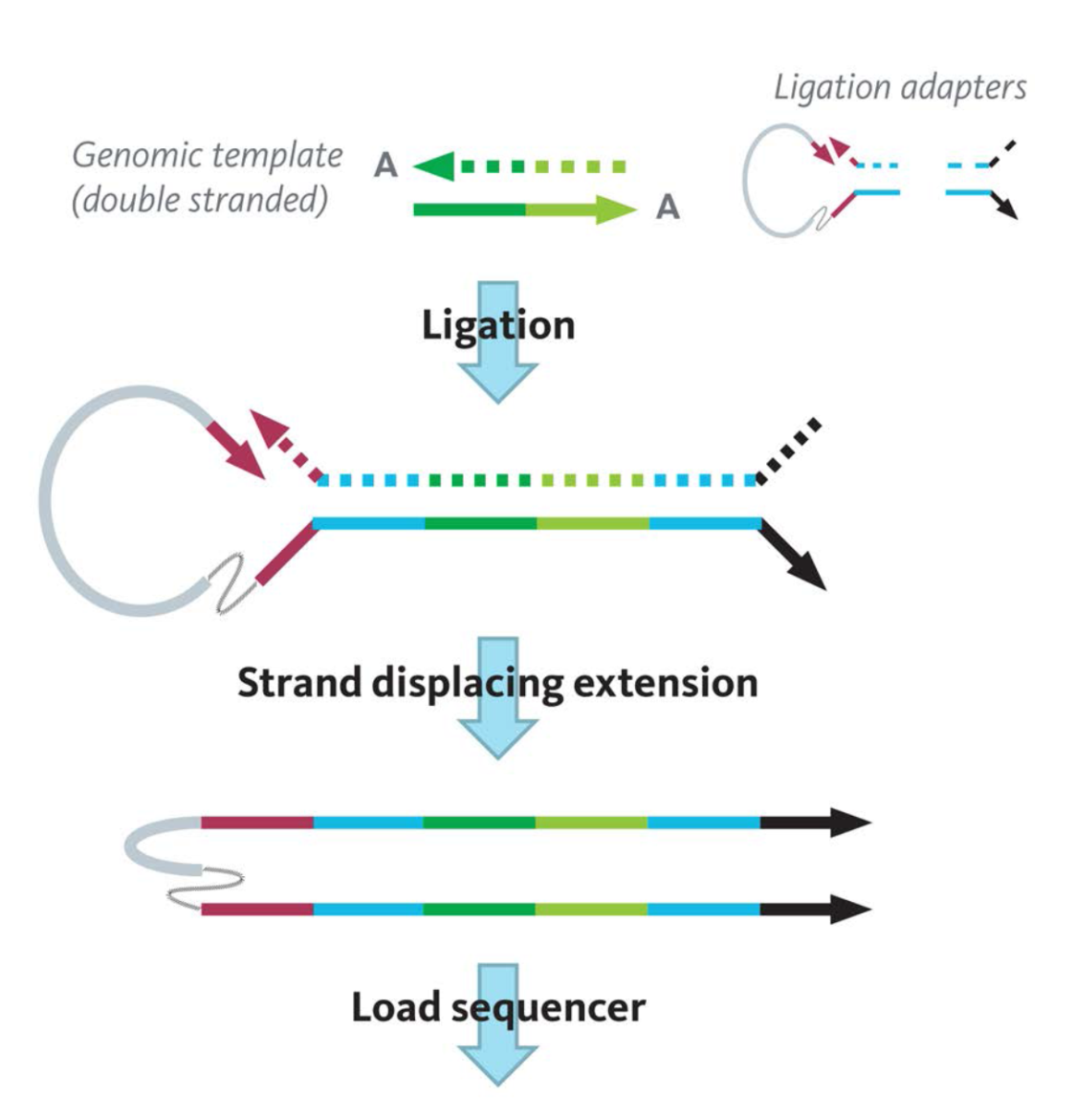

Supplement: S1 Fig — In brief, double-stranded DNA is ligated with unique PEG-linked ‘loop adapters’. The bound priming site on the loop adapter undergoes a single extension with a strand displacing polymerase to generate a molecular construct where each construct and Pro-Seq cluster contains representation from each sense of the starting molecule. After cleanup and quantification, the library is ready to load on the sequencer. This PCR-free workflow is very rapid and can be performed by a single technician in less than four hours (less than two hours hands-on time). Importantly, every linked molecule contains copies from both senses of the starting DNA duplex. (TIF) [file pone.0204265.s004.tif]

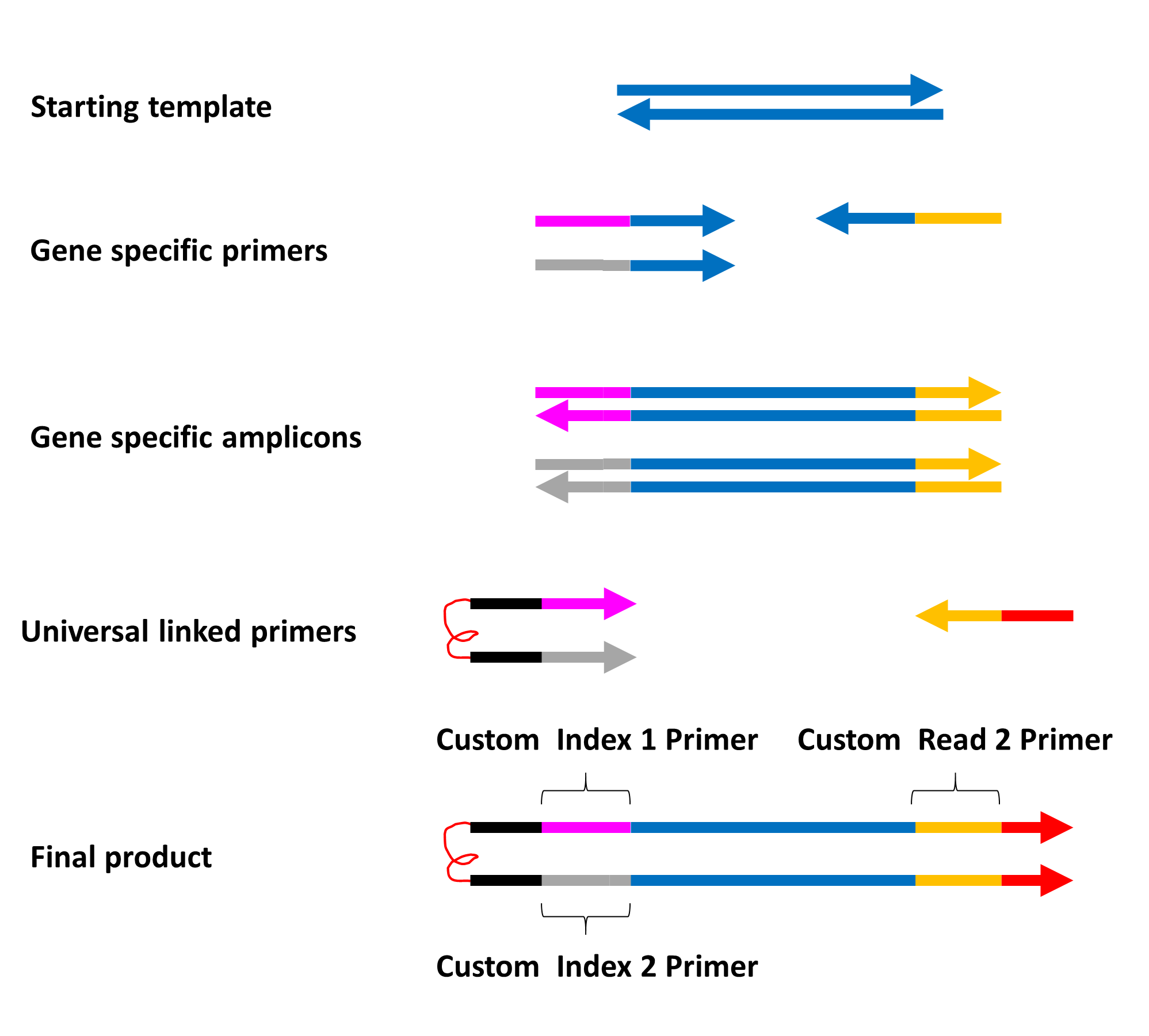

Supplement: S2 Fig — On average, zero or one DNA templates were loaded into each droplet, along with other background DNA (DNA that is not amplified by gene specific primers). Each droplet also contained multiplexed gene specific primers, and universal linked primers. In this work, between seven and 19 amplicons were multiplexed together. Each amplicon used two gene specific forward primers with different linking sequences (pink, grey) to the universal linked primer, which enabled identification of Pro-Seq clusters on the sequencer, along with a single gene specific reverse primer. The two different forward gene specific primers per amplicon created two gene specific amplicon types per target, such that when two linker primers were used, on average both senses of the starting templates were represented in 50% of the Pro-Seq clusters (as the number of linker primers increases, the fraction of clusters representing both senses also increases). Universal 5’ PEG-linked primers containing flow cell adapter sequences (black) extended off the two gene specific amplicons with a single universal reverse primer that contained the second flow cell adapter sequence (red). After sufficient cycling, all universal linkers were ‘filled’ to create the final sequenced product. Not shown is the un-linked reverse complement of the final product which was digested after emulsion breaking, prior to sequencing. Sequencing primer locations were as indicated. (TIF) [file pone.0204265.s005.tif]

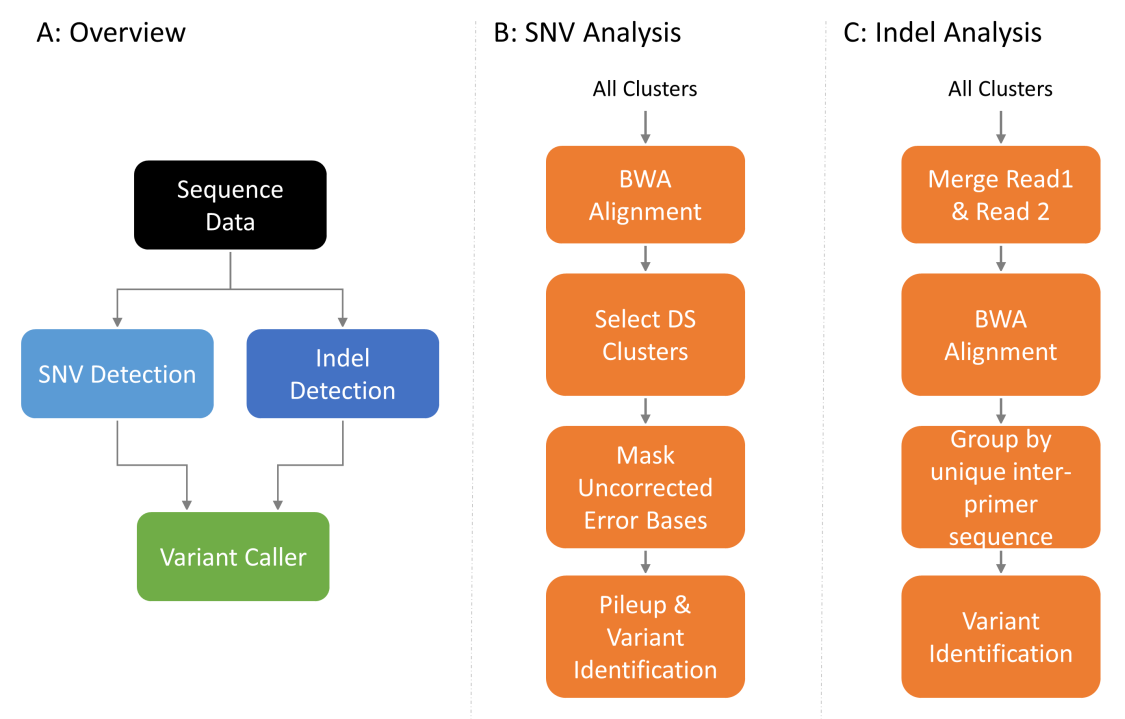

Supplement: S3 Fig — (A) Full analysis overview. SNV and indel detection were handled separately, after which a combined variant caller identified any non-reference sequences. (B) SNV analysis consisted of alignment, doubly-seeded (DS) cluster selection, error base masking (to eliminate remaining errors not corrected during sequencing) and then pileup and variant identification. (C) Indel analysis consisted of alignment, trimming of known primer sequences and grouping by specific inter-primer sequences. Inter-primer sequences were piled up, followed by variant identification. (TIF) [file pone.0204265.s006.tif]

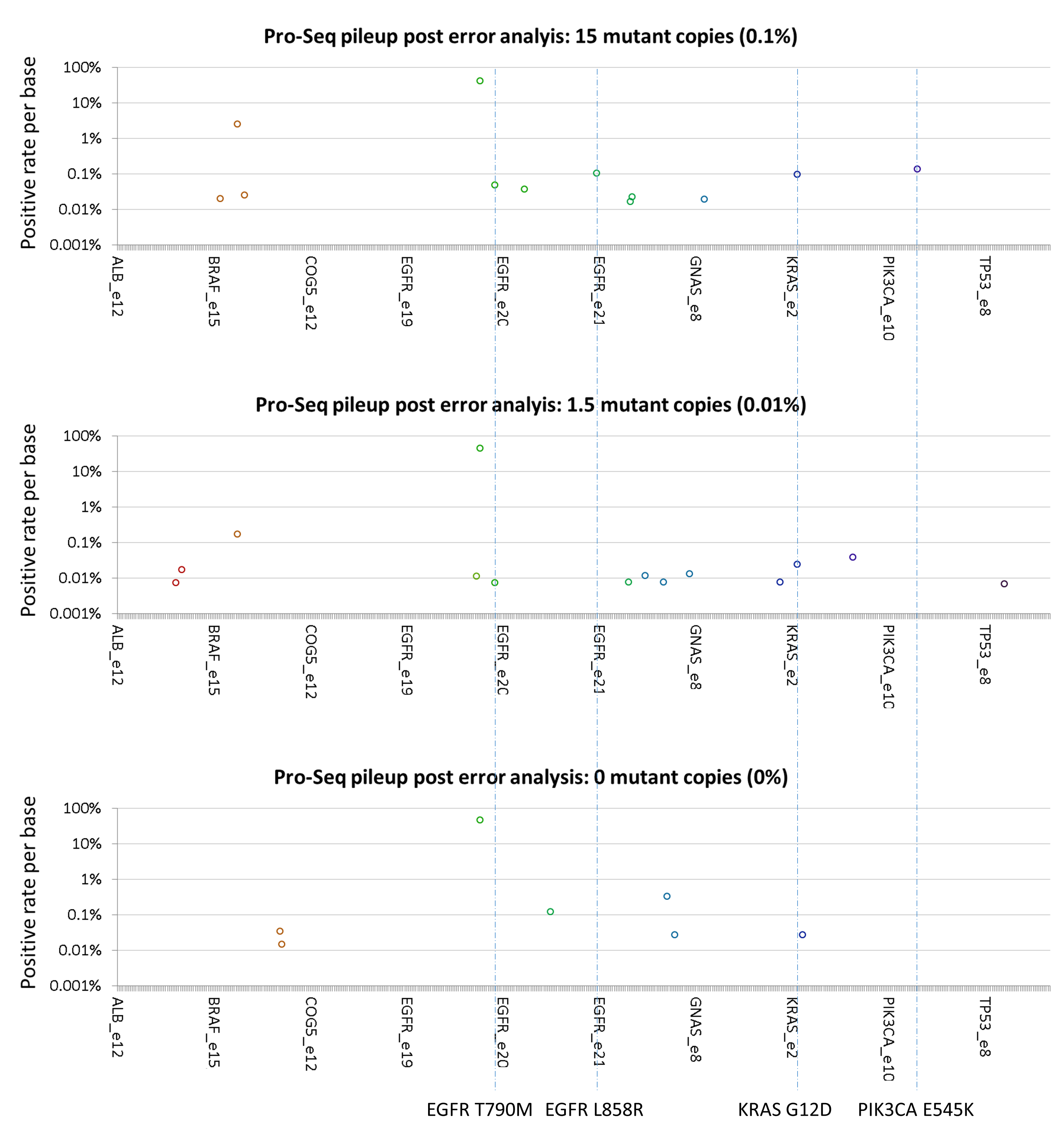

Supplement: S4 Fig — Point mutation pileups for the first replicate of 15, 1.5 and 0 mutant copies shown (top, middle, bottom, respectively), from molecular sensitivity measurements. The background mutations shown in the bottom zero mutant pileup (including the known SNP in EGFR exon 19) may be real mutations present in the plasma of the nominally healthy donor. Other mutations present in the spiked mutant samples (middle, top) may occur in the cell line, consistent with the elevated mutation background found in cell line and described in this manuscript. (TIF) [file pone.0204265.s007.tif]
